# Effects of ASD-associated *daf-18*/PTEN missense variants on *C. elegans* dauer development

**DOI:** 10.17912/micropub.biology.000177

**Published:** 2019-10-16

**Authors:** Carolina González-Cavazos, Mengyi Cao, Wan-Rong Wong, Cynthia Chai, Paul W Sternberg

**Affiliations:** 1 Division of Biology and Biological Engineering, California Institute of Technology

**Figure 1:**
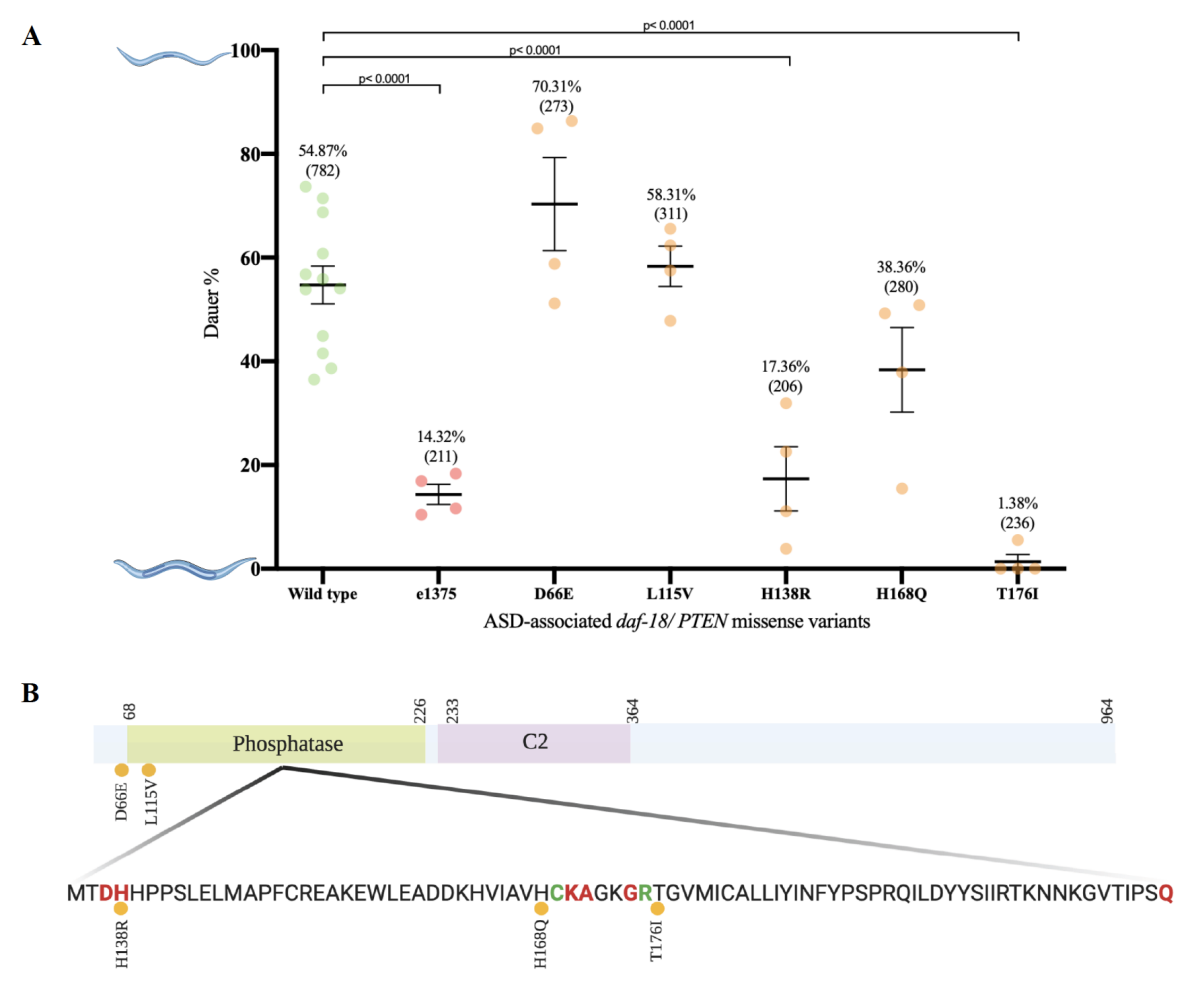
**The effect of *daf-18/PTEN* missense variants on dauer entry.** (A) Percent of dauer-entry in ASD-associated *daf-18*/*PTEN* mutant strains. The dots indicate the percentages of dauer induction of each experiment. The average percentage of dauer-entry of each stain is indicated on the top, and the total worms analyzed are shown in the parenthesis**.** Statistical analysis was performed using one-way ANOVA test followed by multiple comparisons test (Dunnett test). Data are shown in averages and standard errors. (B) Schematic diagram of DAF-18/PTEN. The green box represents the predicted phosphatase domain and the purple box represents the C2 domain. Numbers indicate the corresponding amino acids. Red and green amino acids indicate the active and catalytic sites of the protein, respectively (Conserved Domain Search). The orange dots represent the ASD-associated *daf-*18/*PTEN* missense variants analyzed.

## Description

The relatively simple nervous system and facile genetics of *Caenorhabditis elegans* makes it a potential model organism to study the genetic basis of complex neural diseases, such as autism spectrum disorder (ASD). Our previously published study identified conserved human ASD-missense variants in *C. elegans* orthologs that have a role on morphology, locomotion or fecundity, suggesting *C. elegans* as an efficient phenotypic model to study conserved ASD variants (Wong *et al.*, 2019). One of the ASD-associated genes screened in this study is *daf-18*, an ortholog of *PTEN*. DAF-18/PTEN is a lipid phosphatase protein that dephosphorylates PIP_3_, a critical lipid second messenger that mediates downstream kinase cascade signaling of the insulin pathway, that in turn regulates expression of genes involved in *C. elegans* lifespan and dauer formation (Murphy and Hu, 2013). It is reported that the *daf-18(e1375)* strain, which has a 30–base pairs insertion in the fourth exon, has a dauer defective phenotype (Ogg and Ruvkun, 1998); however, the effect of *daf-18/PTEN* single amino acid substitution in dauer formation has not been evaluated before. Here, we investigate the dauer entry abilities of previously published ASD-associated *daf-*18/*PTEN* missense variants (Wong *et al.*, 2019).

We performed a dauer-entry assay using crude pheromone as a proxy for high population density and heat-killed bacteria as a limited food source. We first determined the crude pheromone concentration required to induce 50% of wild type (N2) strain to enter dauer stage (EC_50_). Using the calculated EC_50_value we evaluated each mutant strain in two independent experiments (Table 1). To control day-to-day variations caused by environmental conditions, N2 controls were used in every trail. Additionally, we used the previously reported *daf-18* dauer-defective strain (*e1375*) as a control (Ogg and Ruvkun, 1998). To analyze the obtained data, we performed one-way ANOVA followed by multiple comparisons test (Dunnett test) (Figure 1A).

**Table 1** Evaluated ASD-associated *daf-18/PTEN* mutant strains.

**Table:** 

Strain name	**PS7439**	**PS7432**	**PS7436**	**PS7430**	**PS7434**
*C. elegans* allele name	*daf-18(sy879)*	*daf-18(sy887)*	*daf-18(sy881)*	*daf-18(sy885)*	*daf-18(sy882)*
*C. elegans* protein change	D66E	L115V	H138R	H168Q	T176I
Human protein change	D22E	L70V	H93R	H123Q	T131I

As expected, we observed statistically significant difference between our wild type and dauer defective control (*e1375)*. Our results show that H138R (PS7436) and T176I (PS7434) *daf-18* mutant strains have a significant difference from the wild type control, and a non-significant difference from the dauer defective control, suggesting these missense alleles cause dauer defective phenotypes to the same level of the *e1375* strain. In contrast, we found the D66E (PS7439), L115V (PS7432) and H168Q (PS7430) mutant strains are significantly different from the dauer defective control and not significantly different from the wild type control, suggesting these three missense alleles do not cause the same level of dauer defective phenotype as the defective control does. Altogether, our current data show that H138R and T176I are defective in dauer entry, while D66E, L115V and H168Q *daf-18* mutant strains are not (Figure 1A).

The Conserved Domain Database (Conserved Domain Search) of NCBI (ID: G5EE01) predicts that four of the five missense variations evaluated in this study are located in the phosphatase domain of DAF-18/PTEN(Figure 1B). It is predicted that amino acid Histidine 138 is located in the active site of the protein, suggesting that H138R (PS7436) amino acid change might have an impact on PIP_3 _substrate specificity, which could explain the dauer defect phenotype observed. Moreover, it is predicted that both amino acid Histidine 168 and Threonine 176 are located close to the catalytic site of the phosphatase domain. We found a dauer defective phenotype resulting from a T176I substitution (PS7434) but not from a H168Q amino acid substitution (PS7430).

These data suggest that certain amino acids in the catalytic and active sites of *DAF-18/PTEN* decrease the function of the protein, in turn regulating *C. elegans* dauer development. Future evaluation of PIP_3 _phosphorylation levels could confirm the effect of these variations on the phosphatase activity. The results of this study contribute to the knowledge of the mechanisms controlling *C. elegans* dauer entry, and to the *C. elegans* phenotype-screening of conserved ASD variations.

## Reagents

Genotyping missense variants

To confirm ASD-associated *daf-18/PTEN* missense variations we genotyped each previously generated strain as follows: about 10 young worms were picked into 5 μL lysis buffer (50 mM KCl, 10 mM Tris pH 8.3, 2.5 mM MgCl_2_) with proteinase K (20 mg/mL; Invitrogen™) and incubated at 65°C for 10 minutes and 85°C for 1 minute. The genomic DNA was amplified by PCR reaction and then treated with a restriction enzyme (New England Biolabs® Inc.) as described in Table 2.

**Table:** **Table 2** ASD-associated strains genotyping information.

**Strain**	**Forward primer**	**Reverse primer**	**Enzyme**	**Wild type**	**Mutants**
PS7439	5’-CCCAATGGTTACTCCTCCTC-3’	5’-CCTGTGTTAGTCCTCCTTCAAT-3’	*SacI*	611 bp	202 bp, 409 bp
PS7432	5’-CCCAATGGTTACTCCTCCTC-3’	5’-CCTGTGTTAGTCCTCCTTCAAT-3’	*SacII*	611 bp	210 bp, 401 bp
PS7436	5’-CCCAATGGTTACTCCTCCTC-3’	5’-CCTGTGTTAGTCCTCCTTCAAT-3’	*AvaI*	611 bp	135 bp, 476 bp
PS7430	5’-TGTCCGACCCGTTAGTCTTC-3’	5’-CCACGTAGACACCAATCAACT-3’	*HpyCH4V*	265 bp	55 bp, 210 bp
PS7434	5’-CTGTCCGACCCGTTAGTCTTC-3’	5’-CCACGTAGACACCAATCAACT-3’	*ApaL1*	340 bp	150 bp, 190 bp

Dauer-entry assay on pheromone plates

The crude pheromone used was extracted from exhausted liquid culture medium, resuspended with distilled water, and stored at −20 °C (Butcher *et al.*, 2007). The day before each experiment, NGM pheromone plates were freshly prepared and dried overnight at room temperatureand young *C. elegans* hermaphrodites of each strain were picked and incubated at 25°C overnight. On the day of the experiment, ∼10 young adults of each strain were placed onto each pheromone plate with 2 µL of OP50 and allowed to lay ∼70-80 eggs before being removed. We added 18 µL of heat-killed OP50 to each plate. After 48 hours of incubation at 25.5 °C, dauers and non-dauers were counted on each plate (Lee *et al.*, 2017).
